# Addition of angled rungs to the horizontal ladder walking task for more sensitive probing of sensorimotor changes

**DOI:** 10.1371/journal.pone.0246298

**Published:** 2021-02-05

**Authors:** Jaclyn T. Eisdorfer, Michael A. Phelan, Kathleen M. Keefe, Morgan M. Rollins, Thomas J. Campion, Kaitlyn M. Rauscher, Hannah Sobotka-Briner, Mollie Senior, Gabrielle Gordon, George M. Smith, Andrew J. Spence

**Affiliations:** 1 Department of Bioengineering, Temple University, Philadelphia, Pennsylvania, United States of America; 2 National Eye Institute, National Institutes of Health, Bethesda, Maryland, United States of America; 3 Lewis Katz School of Medicine, Temple University, Philadelphia, Pennsylvania, United States of America; 4 Shriners Hospitals Pediatric Research Center, Philadelphia, Pennsylvania, United States of America; Szegedi Tudomanyegyetem, HUNGARY

## Abstract

One method for the evaluation of sensorimotor therapeutic interventions, the horizontal ladder walking task, analyzes locomotor changes that may occur after disease, injury, or by external manipulation. Although this task is well suited for detection of large effects, it may overlook smaller changes. The inability to detect small effect sizes may be due to a neural compensatory mechanism known as “cross limb transfer”, or the contribution of the contralateral limb to estimate an injured or perturbed limb’s position. The robust transfer of compensation from the contralateral limb may obscure subtle locomotor outcomes that are evoked by clinically relevant therapies, in the early onset of disease, or between higher levels of recovery. Here, we propose angled rungs as a novel modification to the horizontal ladder walking task. Easily-adjustable angled rungs force rats to locomote across a different locomotion path for each hindlimb and may therefore make information from the contralateral limb less useful. Using hM3Dq (excitatory) Designer Receptors Exclusively Activated by Designer Drugs (DREADDs) expressed in large diameter peripheral afferents of the hindlimb in the intact animal, we characterized the sensitivity of our design to detect stepping differences by comparing locomotor changes observed on angled rungs to those observed on a standard horizontal ladder. On our novel asymmetrical ladder, activation of DREADDs resulted in significant differences in rung misses (p = 0.000011) and weight-supporting events (p = 0.049). By comparison, on a standard ladder, we did not observe differences in these parameters (p = 0.86 and p = 0.98, respectively). Additionally, no locomotor differences were detected in baseline and inactivated DREADDs trials when we compared ladder types, suggesting that the angled rungs do not change animal gait behavior unless intervention or injury is introduced. Significant changes observed with angled rungs may demonstrate more sensitive probing of locomotor changes due to the decoupling of cross limb transfer.

## Introduction

Sensitive analysis of sensorimotor function is essential to evaluate potential therapeutic interventions. There are several methods to measure locomotor recovery in rodents, including the Basso, Beattie, Bresnahan (BBB) Open Field Locomotor Scale [1], the horizontal ladder walking task [2–4], gait analysis [5–7], kinematic analysis [8], the rotarod [9, 10], grid walk [11], beam walking tests [12], and force platform/arena methods [13]. The horizontal ladder walking task is advantageous as it was designed for rapid analysis of skilled walking, limb placement, and limb coordination after disease or injury, such as spinal cord injury [2]. However, this task is limited to evaluation of gross differences in locomotion and thus small changes may be undetectable [2–4]. This may be a result of a neural compensatory mechanism known as “cross limb transfer”, or the contribution of the contralateral limb to estimate an injured or perturbed limb’s position [14]. The robust locomotion involved in this phenomenon may obscure subtle locomotor outcomes that are evoked by clinically relevant therapies (such as genetic neural manipulation), in the early onset of disease, or between higher levels of recovery. Thus, it is crucial to develop more sensitive sensorimotor assays to better quantify subtle changes in locomotion due to potentially precise, subtle changes in neuromotor systems.

The horizontal ladder walking task evaluates forelimb and hindlimb function deficits after disease or injury [2, 4]. It was first developed to assess movement dysfunction after sensorimotor cortex injury in rats [2, 15], but has since also been used for evaluation of spinal cord injury, traumatic brain injury, poststroke, etc. [15–17]. In the task, animals are directed to run in one direction across a horizontal ladder. There are several distinct stepping or foot-fault events that can occur: foot was placed on rung with full body weight support: “Hit”; foot was placed on rung, but slipped off: “Slip”; foot did not touch rung at all: “Miss” [2].

Conventionally, a horizontal ladder is made of parallel rungs that are either placed at equal or unequal distances apart [2]. In both equally and unequally spaced parallel rung arrangements, both hindpaws have the same locomotion path. Locomotion path is defined here (and throughout) as the footfalls of each hindpaw as the animal crosses the ladder. While the placement of rungs in a parallel fashion can indeed detect neurological deficits if severe enough, this arrangement is limited by the cross limb transfer phenomenon. As such, parallel rungs cannot control for compensatory effects originating from the contralateral limb, an occurrence which may conceal subtle sensorimotor deficits.

In 1894, Edward Wheeler Scripture first introduced the concept of “cross limb transfer”, or the effects of one limb on its contralateral pair [18]. Cross limb transfer (used here and throughout) is also commonly referred to as cross education, cross training, or cross exercise. It is well established that both contralateral and ipsilateral cortical motor areas display increased excitability during a unilateral movement, but the mechanism driving this occurrence is debated [19–21]. There are two prevailing hypotheses that explain dual hemisphere activation during unilateral movement. The bilateral access hypothesis states that motor engrams developed in the hemisphere of one limb are accessible to the motor networks of both limbs via the corpus callosum during task performance and for making adjustments during a task. The cross activation hypothesis states that supraspinal centers exhibit bilateral activation and adaptation even if a task is performed unilaterally [22]. In both hypotheses, unilateral tasks can elicit changes in motor circuit organization and this information can be used by both the ipsilateral and contralateral limbs.

A spinal component to cross limb transfer that can be activated while a task is being performed has also been previously demonstrated. Studies with electrical stimulation report that muscle contraction can increase spinal excitability in the homologous muscle on the contralateral side [23, 24]. Both endogenous proprioception and cutaneous afferent input exert strong excitatory effects on contralateral motor neurons during this occurrence [14]. Since the anatomical basis of the nervous system is bilaterally symmetrical with crossed representation, the development of a technology that can isolate limb performance from compensation or guidance transferred from the contralateral limb can provide useful information on spinal circuit changes that accompany unilateral limb perturbation, neuromodulation, or injury.

Although modifications have been made to horizontal ladders [3, 4], to our knowledge, none were designed to uncover any changes that may be concealed by the occurrence of cross limb transfer. We thereby sought to develop a more sensitive ladder assay by decoupling this phenomenon via the introduction of different locomotion paths to contralateral limb pairs. Thus, the central contribution of this paper is a novel ladder with angled rungs to more sensitively probe for changes in sensorimotor control. For the purpose of this paper, we refer to our novel asymmetrical ladder with angled rungs as “asymmetrical” henceforth. Our asymmetrical ladder is capable of being set to a multitude of variable distances and angles between rungs. This introduces a high number of possible arrangements.

To evaluate whether this design had improved detection sensitivity for small changes in sensorimotor control, we utilized the genetic tool Designer Receptors Exclusively Activated by Designer Drugs (DREADDs) to modulate afferent feedback in intact, wild-type, freely moving rats. Importantly, DREADD technology was chosen to validate our design as its subtle effects in the intact animal may be concealed by cross limb transfer. We introduced hM3Dq (excitatory) DREADDs into the right lumbar dorsal root ganglia (DRG) and hypothesized that angled rung arrangements would more sensitively discriminate DREADDs-evoked hindlimb locomotor changes. Upon activation by its receptor-ligand clozapine-N-oxide (CNO), hM3Dq DREADDs enhance neuronal firing by activating Gq signaling pathways [25] and, as such, may influence muscle recruitment when expressed in afferents of the periphery. In this study, we compared performance on angled rungs to symmetrical rungs. We chose to compare our design to equally-spaced parallel rungs as this is complementary to published studies with the ladder task and DREADDs activation [26, 27].

Based on simulation studies that find activation of afferents should extend joints [28], we hypothesized that targeting the lumbar DRG would result in increased foot-fault events by extending the muscles about the hip. We report here the measured rate of correct and incorrect footfall placements whilst animals locomoted over standard symmetrical and our asymmetrical ladders with and without activation of hM3Dq DREADDs. Results from our asymmetrical ladder demonstrate ease of rung adjustments between trials to introduce new locomotion paths quickly. Results further reveal locomotor deficits that were not observable with a standard symmetrical rung arrangement, which may be a result of the decoupling of the cross limb transfer compensatory mechanism.

## Materials and methods

### Subjects

This study was carried out in strict accordance with the recommendations in the Guide for the Care and Use of Laboratory Animals of the National Institutes of Health. The protocol was approved by the Institutional Animal Care and Use Committee (IACUC) of Temple University (Protocol No.: 4675 awarded to Dr. Andrew J. Spence). All surgical procedures were performed under aseptic conditions. Animals were anesthetized with ketamine and xylazine, and all efforts were made to minimize suffering. Eight female Sprague-Dawley rats (200–250 g at the start of the study) were obtained from Charles River Laboratories Inc. (Wilmington, Massachusetts). Animals were housed in pairs with access to food and water *ad libitum* in holding rooms that were maintained on a 12-hour light/dark cycle. Experiments were conducted during the light phase. One animal showed signs of neurological injury, which is a predetermined humane endpoint, and was subsequently humanely euthanized.

### Ladder design

The components and overall design of the novel rat ladder system were modeled in computer-aided design (CAD) software (Fusion360, AutoDesk, San Rafael, CA). Several critical functional characteristics were implemented in the design. These included the use of bolt mounted ladder rungs that could be slid inside of the tracks for repositioning, the oversizing of the track runs relative to the bolts to enable multiple angles to be set for each rung, the staggered overlap of the track runs to provide stability, the use of transparent components when possible to aid visualization and computational tracking, the inclusion of measurement marks, and the design of a wall tensioning device to allow straightening of the walls relative to the locomotion path across the horizontal ladder. The tracks on which the bolts would slide were designed in 2D CAD (AutoCAD, AutoDesk, San Rafael, CA) for laser cutting applications (S1 Appendix). 3D renderings of the asymmetrical ladder are available in S2 and S3 Appendices.

### Ladder fabrication

**Fig 1:**
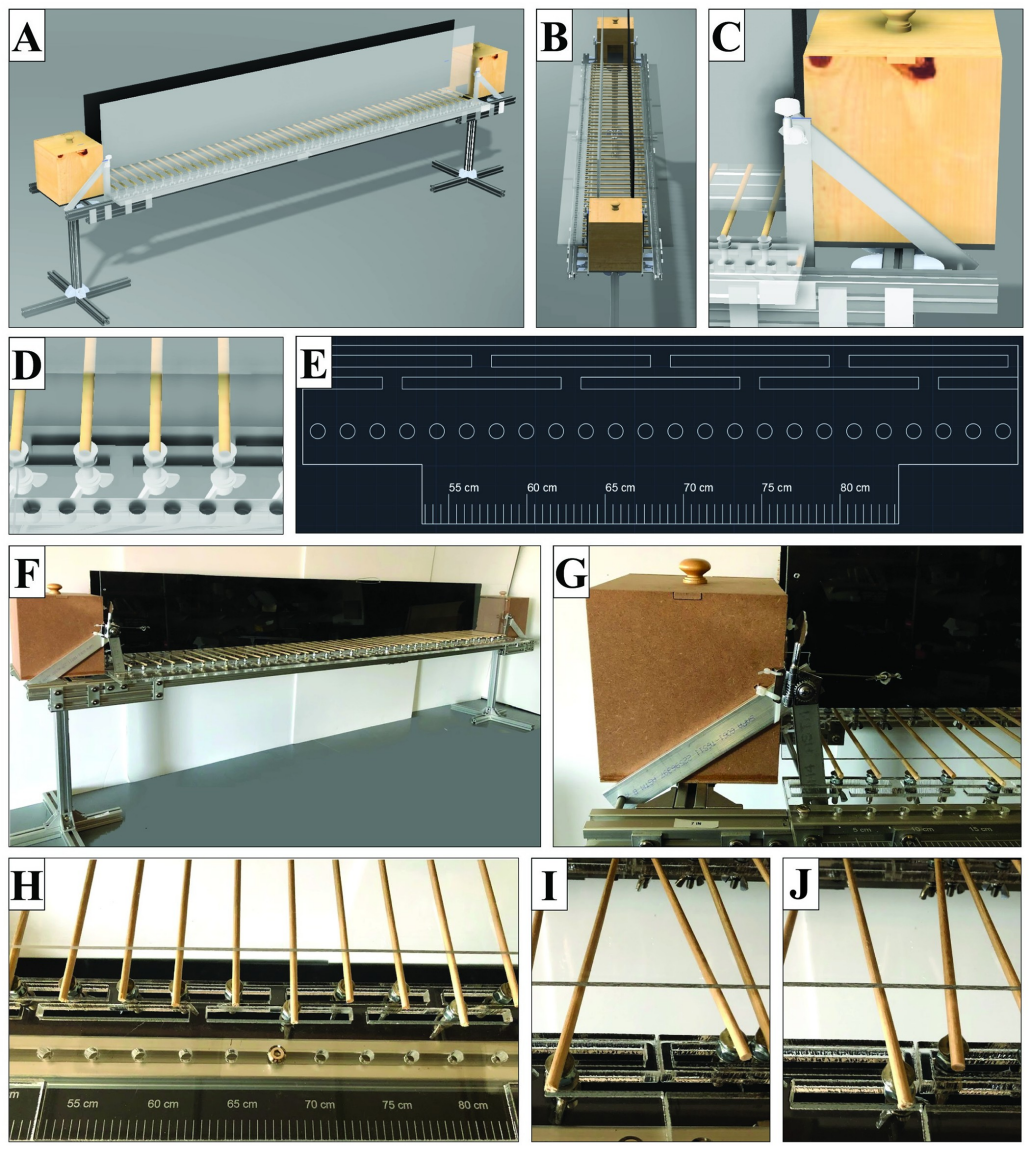
CAD and construction of the modular asymmetrical ladder. (A) A CAD (computer-aided design) rendering of the complete ladder. (B) A longitudinal projection of the CAD rendering. (C) A close-up CAD rendering of the wall tensioning mechanism. (D) A close-up CAD rendering of the rung adjustment mechanism. (E) A 2D CAD image used for laser cutting of the rung adjustment mechanism. (F) The actual complete ladder after fabrication. (G) A close-up of the actual wall tensioning mechanism. (H) A close-up of the actual rung adjustment mechanism with etched measurement marks. (I, J) A demonstration of the adjustment and angling of the ladder rungs.

The transparent positioning tracks were fabricated from 3/8” ACRYLITE^®^ cast acrylic (Evonik Industries, Essen, Germany) and cut with a VLS 6.60 laser cutting system (Universal Laser Systems, Scottsdale, AZ). The primary frame of the ladder was constructed with 30–3030 T-slot aluminum extrusion (ZYLtech, Spring, Texas) affixed with M6 bolts and 30 mm corner brackets. Rungs were constructed by using 2 part epoxy (Gorilla Glue, Sharonville, OH) to affix 3/16” wooden dowels cut to 9” (McMaster-Carr, Elmhurst, IL) to 1/4"-20 x 1-1/2” flat head screws (McMaster-Carr, Elmhurst, IL) which were then mounted to the acrylic positioning tracks using wingnuts with oversized washers (McMaster-Carr, Elmhurst, IL). The transparent and opaque walls were constructed with 1/8” plexiglass (Everything Plastics, Philadelphia, PA) and standard 1/8” plywood, respectively. The opaque wall was spray painted black to aid in the visualization of locomotion. The starting and ending containers were constructed with standard 1/8” medium density fiberboard. The tensioning device and other exterior parts were individually prepared on a Bridgeport mill (Hardinge, Inc., Elmira, NY) using 1/16” and 1/8” aluminum flat bar and 1/8” aluminum bar (McMaster-Carr, Elmhurst, IL). Tension was achieved using standard bass tuning pegs. The design of the ladder system was completed in computer-aided design (CAD), including a complete isometric view (Fig 1A), longitudinal views (Fig 1B), the design of the tensioning system (Fig 1C), the mechanism of rung placement on the track (Fig 1D), and a portion of the 2D CAD file used to laser cut the acrylic track (Fig 1E). Using these CAD files, the entire ladder system (Fig 1F) and the tensioning device (Fig 1G) were fabricated. Rungs were affixed to the acrylic tracks for feasible locomotion (Fig 1H). Rungs can be repositioned to change location and angle (Fig 1I and 1J). The completed ladder was confirmed to have the structural integrity to support rat locomotion without slipping or loosening of the rungs and that sufficient rungs can be added to the system to have a no greater than 6 cm maximum distance between one another. The complete ladder has an overall runnable length of 182 cm and a width of 10 cm.

### Measurement of ladder rung gaps and angles

To evaluate the gap distances and angles between rungs on the two ladder types, images were taken from set heights above each ladder and processed in ImageJ [29]. Using in-built scale markers, the largest and smallest distances were measured between each rung using the “measurement” function. Angles were measured using the angle tool. A summary of those results including average, maximum, and minimum are shown in S1 Table. The gaps between rungs on the asymmetrical ladder ranged from approximately 1.75 to 5.5 cm with a maximum angle varying from 81 degrees to 98 degrees from perpendicular. The average gaps between rungs was 3.579 cm and 90.583°.

### DRG injections

All surgical procedures were carried out under aseptic conditions. Lumbar dorsal root ganglia (DRG) L2 through L5 were chosen to be injected with the hM3Dq DREADDs viral construct as these DRG are reported to innervate muscles about the hip and throughout the leg [30–32]. Adeno-associated virus serotype 2 (AAV2) was selected as a viral vector to deliver DREADDs as this virus primarily targets large diameter afferents when directly injected into the DRG [33, 34]. By extension, we intended to increase excitation of large diameter peripheral afferents as these neurons are thought to largely influence recovery after injury, e.g. spinal cord injury [35–37]. All viral constructs utilized the human synapsin (hSyn) promotor and also encoded an mCherry fluorescent tag for postmortem histology. Animals were anesthetized with a mix of Ketamine (100 mg/mL, Zetamine, Vet One, Boise, ID), Xylazine (100 mg/mL, AnaSed, Lloyd Laboratories, Shenandoah, IA), and sterile saline injected intraperitoneally and maintained at this level with supplemental doses. For DRG exposure, an approximately 5 cm incision was made in the skin along the dorsal midline starting from the first lumbar (L1) vertebrae. Superficial muscular fascia were incised and paraspinal muscles were separated via dissection to expose the lateral surface of the right L2 to L5 vertebrae as well as the dorsal surface of the medial portion of their transverse processes. The accessory processes that descend from the L2 through L5 vertebrae were removed using a 1 mm rongeur (Friedman bone rongeurs, Fine Science Tools). Laminar bone was removed using the same rongeur to expose the distal third of the DRG. Fascia covering the DRG were removed with 0.1 mm ultra-fine clipper scissors (Fine Science Tools, catalogue number: 15300–00). Once DRGs were exposed, animals were attached to stereotactic spinal clamps for injections. pAAV-hSyn-DIO-hM3D(Gq)-mCherry (Addgene plasmid #44361; Roth, 2016) was co-injected with scAAV-Cre (generously gifted to us by the Hu Lab [38]) and Fast Green FCF (#F7258, Sigma-Aldrich), a method that was adopted in part from Gompf et al. [39]. Four right DRG were injected with (excitatory, n = 7) DREADDs per animal using a micromanipulator. Each DRG received 1 uL pAAV-hSyn-DIO-hM3D(Gq)-mCherry at a flow rate of 20 nL/s. Following injection, the pipette tip was left in place for 5 min for distribution of fluid and equalization of tissue pressure. Musculature and skin were closed with 4–0 chromic gut sutures (DemeTECH, Miami Lakes, FL) and surgical wound clips, respectively. After surgery, animals were administered 10 cc sterile saline. Cefazolin (0.5 g Cefazonlin powder reconstituted in sterile saline, Cat. No. NDC #0143-9923-90, Hikma Pharmaceutical USA, Inc., Eatontown, NJ) and analgesic (Rimadyl, 1 mg tablet, Cat. No. MD150-2, Bio-Serv, Flemington, NJ) was also given after surgery and for two additional days thereafter.

### Hargreaves test (thermal hyperalgesia)

To ensure DREADDs did not transfect thermal nociceptors, animals were subjected to the Hargreaves test (Ugo Basile, catalog number: 37370) with methods described by Goh et al. [40]. In brief, rats were placed individually in clear plastic chambers for 5 min each day for three days to acclimate. Paw withdrawal latency times were then recorded for the right hindpaw in biological triplicates at a heat stimulation of infrared intensity 90. Measurements were obtained prior to DRG injections (baseline), after DREADDs expression without CNO administration (-CNO), and DREADDs activation with 4 mg/kg dosage of CNO injected intraperitoneally (+CNO). In the +CNO condition, measurements were recorded between 30–60 min after CNO injection.

### Video recording

Animals were trained for one week prior to the first video recording. For the first training session, animals were acclimated to the horizontal ladder walking task whereby they were allowed to move freely across the horizontal ladder. The following training sessions involved directed running of the animal across the ladder from one neutral holding chamber to the other on the distal end of the ladder. For video recording, a camera (Hero 7, GoPro) was placed in the center of the horizontal ladder at an angle perpendicular to the ladder and recorded at 120 frames per second. Each video recording consisted of four successful ladder crossings. All animals were recorded crossing the ladder in the same direction with the right hindlimb facing the camera. Prior to DRG injections of hM3Dq DREADDs, baseline horizontal ladder scores were recorded for all animals. Animals were then tested for injury after DRG injection surgery with methods described by Fischer et al. [41].

DREADDs achieve putative maximal expression approximately 3 weeks after injection [25, 42], which is when we began examination of locomotion across horizontal ladders with and without CNO administration (-CNO and +CNO). We compared the ability to detect locomotion differences of our asymmetrical ladder with angled rungs to a horizontal ladder with equally-spaced parallel rungs [26, 27]. A maximum of 6 cm between any two points on adjacent rungs was employed to ensure feasible locomotion across standard symmetrical and asymmetrical rung arrangements. If rungs were placed >6 cm apart, animals would consistently fall through the rungs. The minimum distance between rungs that we employed was approximately 1.8 cm, which was the intrinsic limits of our design. We observed normal stepping patterns, with animals placing their paws on every rung, when the rungs were placed approximately 1.8 cm apart. A full characterization of rung distances is provided in S1 Table. Video recordings of animal locomotion with hM3Dq DREADDs expression (-CNO) and activation (+CNO) were obtained on different days to prevent variability caused by fatigue. Furthermore, DREADDs expression (-CNO) was recorded before CNO administration (+CNO) to limit synaptic changes that may arise from increased neural excitation. DREADDs activation (+CNO) was recorded with intraperitoneal injections of CNO at a dosage of 4 mg/kg. All tests involving CNO were started no earlier than 30 minutes after injection and ceased no later than 60 minutes after injection to ensure maximal DREADDs activation. Asymmetrical rung arrangements were changed to prevent learning of rung patterns during repetitive trials [43] and locomoting animals were examined in real-time to ensure animals were not leaning against the walls as they were crossing the ladder. Only trials where the animal used the midline of the runway were analyzed.

### Horizontal ladder task analysis

Recordings were analyzed in terms of right hindlimb placement on a rung (full weight bearing stepping and slipping off the rung) or misplacement between rungs. There were three types of trials: before DRG injections: “Baseline”; DREADDs expression without CNO administration: “-CNO”; and DREADDs activation by intraperitoneal injection of 4 mg/kg dosage of CNO: “+CNO”. Observers were blind to the type of trial and scored each animal in an Excel spreadsheet. Using methods previously described [2, 44], we observed one distinct stepping event and two distinct foot-fault events: foot was placed on rung with full body weight support: “Hit”; foot was placed on rung, but slipped off: “Slip”; foot did not touch rung at all: “Miss.” The right hindlimb was scored with a distinct footfall event (Hit, Slip, and Miss) in completed trials (4 traversals across the ladder). Number of footfall events were calculated as a percentage out of total steps (ranging from 56 to 68 steps) as a means of standardization.

### Training of scorers

Individuals were trained before they were permitted to score independently. Training consisted of randomly assigning a scorer five videos of animals crossing either the symmetrical or asymmetrical horizontal ladders. Each video had an average of 62 steps, with a range of 56 to 68 steps. Once individuals achieved greater than 95% match to a concealed “answer key” generated by the experiment designers in all five of the videos they scored, they then were authorized to score independently. Scorers were blind to type of trial (Baseline, -CNO, +CNO) and all videos were scored in slow motion to resolve each hindlimb placement event.

### Immunohistochemistry

When the study concluded, expression of hM3Dq DREADDs in large diameter peripheral afferents was characterized in postmortem histological analyses by amplifying mCherry fluorescent tags. Animals were euthanized with lethal overdoses of Fatal-Plus (Cat. No. V.P.L. 9373, Vortech, Dearborn, MI) and intracardially perfused with 4% paraformaldehyde. Injected DRG and corresponding spinal cords were grossly dissected and post-fixed for 24–48 hours (4°C). The tissue was then transferred to 30% sucrose dissolved in phosphate-buffered saline (PBS) for 3–5 days. DRG and spinal cord were frozen and mounted separately. Using a cryostat, 10 μm DRG sections and 20 μm coronal spinal cord sections were affixed to Colorfrost Plus microscope slides (Cat. No. 12-550-18, Fisher Scientific, Hampton, NH). Slide-fixed sections were washed with phosphate-buffered saline tween (PBS-T) 3 times. To amplify the mCherry signal, sections were incubated with primary antibody dsRed (rabbit, polyclonal; Cat. No. 632496, Takara Bio Inc., Mountain View, CA) at 1:400 overnight at 4°C. Sections were then washed in PBS-T 5 times and incubated with secondary antibody Alexa Fluor 594 (donkey anti-rabbit; Cat. No. 111-585-144, Jackson Immunoresearch Laboratories Inc., West Grove, PA) at 1:400 for 2 hours at room temperature. After washing with PBS-T 5 times, sections were air-dried and cover-slipped with Fluoromount-G (Cat. No. 0100–01, VWR International, Radnor, PA). Fluorescent Nissl NeuroTrace, Cat. No. N21479, ThermoFisher Scientific, Waltham, MA) at 1:400 was used to visualize all neurons of the DRG with methods provided by the manufacturer.

Images were acquired using a Zeiss microscope (Jena, Germany) at 10x magnitude and stitched together using Adobe Photoshop. To obtain hM3Dq DREADDs transfection rate within an injected DRG, DRG sections that were 10 μm apart were stained with either dsRed or Fluorescent Nissl. The ratio of dsRed (hM3Dq DREADDs) positive cells to total neurons of the DRG (Fluorescent Nissl positive cells) was obtained using ImageJ.

### Statistical approach

To account for differences in number of steps taken per trial (four traversals), the proportion of hits, misses, and slips was calculated according to the total number of recorded steps. In data containing proportions, resultant values always land between 0 and 100 percent. This skews the variance of the data and risks invalidating evaluation of statistical significance [45]. As described by Sokal and Rohlf [45], we applied an arcsine transformation to remove the variance from the means of our data, enabling statistical analysis. After arcsine transformation, one-way ANOVA and Tukey multiple comparisons tests were performed on the data. Animals were analyzed across the three types of trials: pre-DRG injections: “Baseline”; DREADDs expression without CNO administration: “-CNO”; and DREADDs activation by CNO (4mg/kg): “+CNO”. A p-value < 0.05 was considered significant. Data is presented as mean ± standard error of the mean (SEM). Comparisons in which both groups would have a standard deviation of zero were not evaluated.

## Results

### Characterization of DREADDs expression

**Fig 2:**
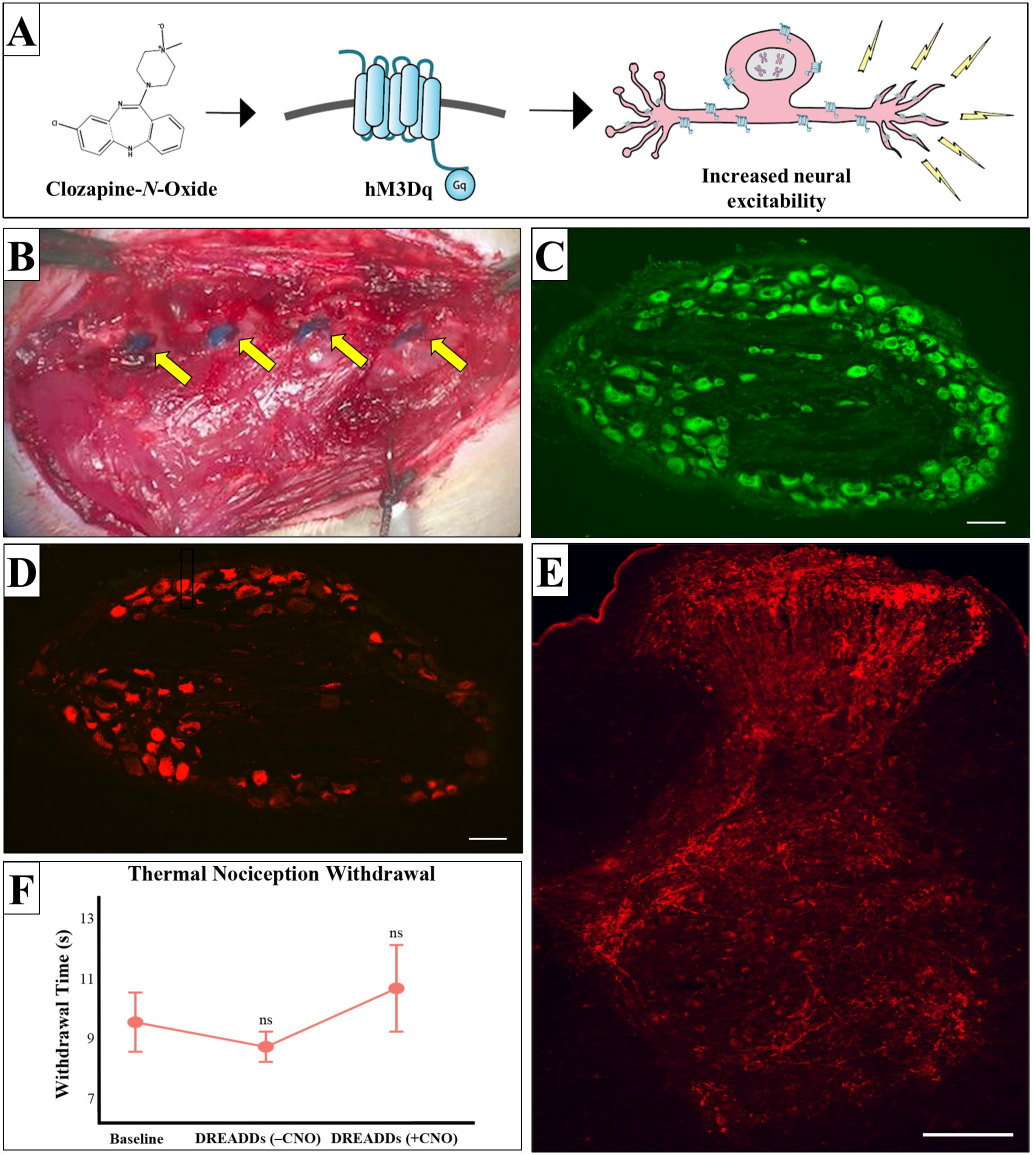
Injection of Designer Receptors Exclusively Activated by Designer Drugs (DREADDs) into rat L2-L5 DRG. (A) Schematic of hM3Dq (excitatory) DREADDs. DREADDs are a transmembrane protein activated by its chemical actuator clozapine-N-oxide (CNO). Potential downstream neuronal effect of hM3Dq DREADDs is increased neuronal excitation. (B) Photograph of L2-L5 DRG injection in wild type Sprague Dawley rats (from right to left; yellow arrows). Injected DRGs (blue) are visualized with Fast Green FCF. (C, D) Immunostaining shows we achieve approximately 42% DREADDs transfection in rat DRGs. Fluorescent Nissl shows total DRG neurons (green; C) and dsRed amplified mCherry in virally transfected neurons (red; D). C and D are sections that are 20 um apart. Scale bar, 100 um. (E) Immunostaining in corresponding lumbar spinal cord of DREADDs injected DRGs. DREADDs positive axons can be observed extending throughout the dorsal and ventral horn. Scale bars 200um. (F) Hargreaves paw withdrawal response times to thermal nociception activation of rats before DRG injection (Baseline), after DREADDs expression without CNO administration (–CNO), and DREADDs expression with 4 mg/kg dosage of CNO (+CNO). Data were analyzed by one-way ANOVA and Tukey multiple comparisons tests and expressed as mean ± SEM. We did not observe any significant changes to paw withdrawal response time after DREADDs were expressed (–CNO; 8.7 s ± 1.24 s; p = 0.85; n = 7 rats), and when DREADDs were activated (+CNO; 10.65 s ± 3.55 s; p = 0.41; n = 7).

Once activated by CNO, hM3Dq (excitatory) DREADDs act to increase neuronal excitability by depolarizing expressing cells (Fig 2A). On average, we observed a DREADDs transfection rate of 42% in L2-L5 (lumbar) DRG (Fig 2B–2D). Corresponding spinal cord sections show transfected cells project throughout the ventral and dorsal horn (Fig 2E). We further report no significant changes to thermal nociceptive paw withdrawal response time between Baseline (pre-DRG injection; 9.52 s ± 2.42 s; mean ± SD; n = 7 rats; and hereafter unless otherwise mentioned), when DREADDs were expressed (-CNO; 8.7 s ± 1.24 s; p = 0.85; n = 7; one-way ANOVA and Tukey multiple comparisons tests and hereafter unless otherwise mentioned), and when DREADDs were activated (+CNO; 10.65 s ± 3.55 s; p = 0.41; n = 7). These data suggest nociceptive afferents are not considerably affected by DRG injections or by DREADDs excitation (Fig 2F).

### Ladder walk validation

**Fig 3:**
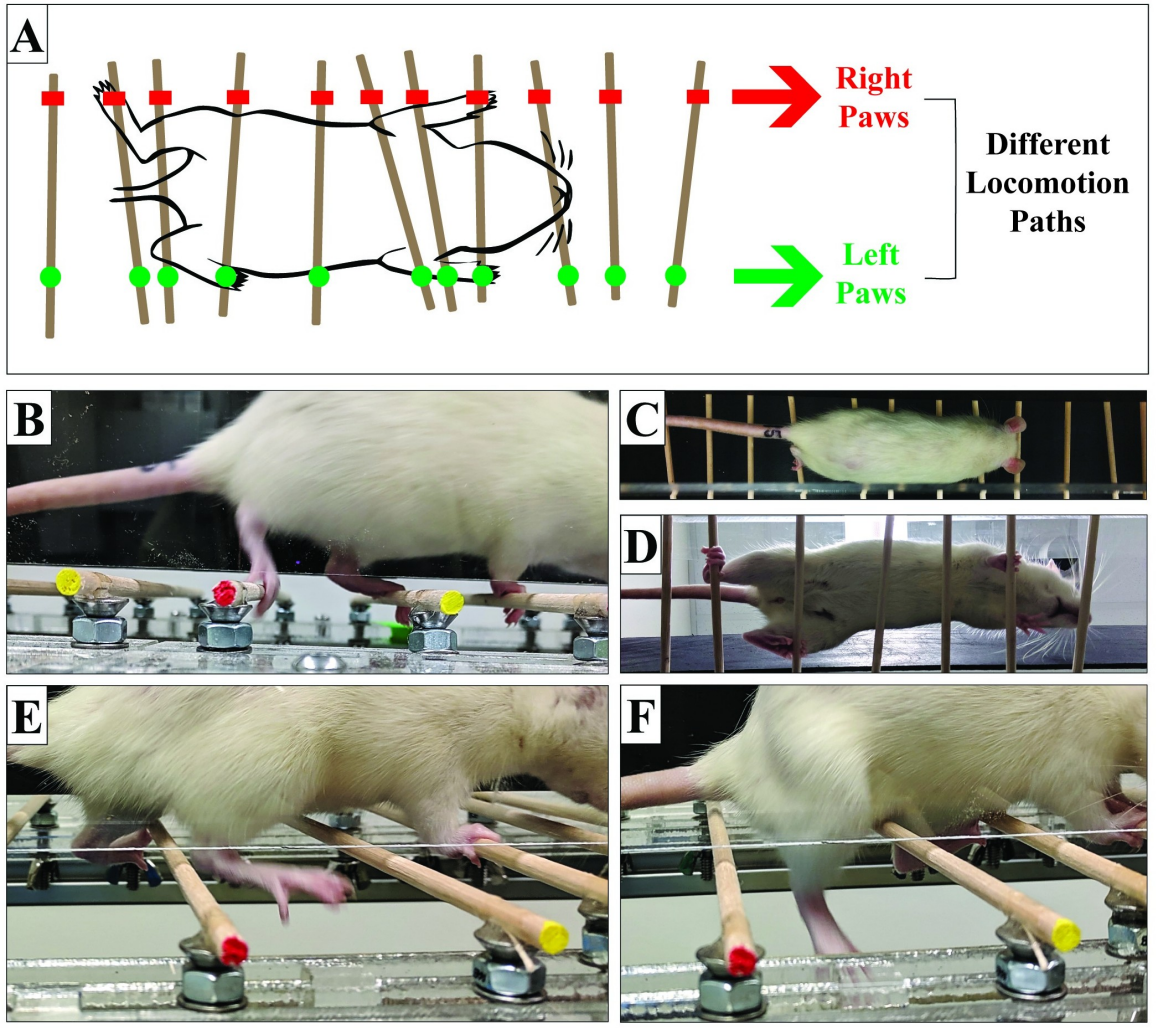
Rat locomotion on horizontal ladder walking task. (A) Illustrative rat locomotion from bottom view on novel asymmetrical ladder. (B-D) Rat walking with full weight bearing (e.g. “Hit”) from lateral view (B), top-down view (C), and bottom view (D). (E, F) Rat during a foot-fault event (e.g. “Miss”).

On symmetrically arranged rungs, animals had the same locomotion paths for contralateral limb pairs, whereas on angled rungs, they were different (Fig 3A). All hindlimb footfalls were visible in video recordings (Fig 3B) and animals did not lean against the plexiglass as they were crossing the horizontal ladder (Fig 3C and 3D).

### Intra-ladder comparison

All raw ladder data obtained from trained scorers and calculations performed on these data are available in S4, S5 Appendices, and S2 Table. All hit, slip, and miss data is presented as arcsine transformed data.

To evaluate the sensitivity of our ladder as it would be employed in research applications, we performed an ANOVA analysis between different treatment conditions of rats run on the same ladders (i.e. asymmetrical ladder before and after administration of CNO). Henceforth, we refer to this analysis as “intra-ladder” comparison. Significant differences were not detected for hits, misses, or slips in any of the comparisons associated with the symmetrical ladder. Neither was significance detected in the comparisons between the baseline and without CNO administration after DREADDs injections (-CNO) conditions for the asymmetrical ladder. Significance was detected in only two conditions, the asymmetrical ladder before and after the administration of CNO for both hits (asymmetrical (-CNO) hit rate = 1.472 ± 0.126, asymmetrical (+CNO) hit rate = 1.340 ± 0.111; *F* = 10.03; degrees of freedom, *df* = 4; p = 0.049) and for misses (asymmetrical (-CNO) miss rate = 0 ± 0, asymmetrical (+CNO) hit rate = 0.0239 ± 0.0118; *F* = 28.68; *df* = 4; p = 0.000011) (S3 Table).

### Inter-ladder comparison

**Fig 4:**
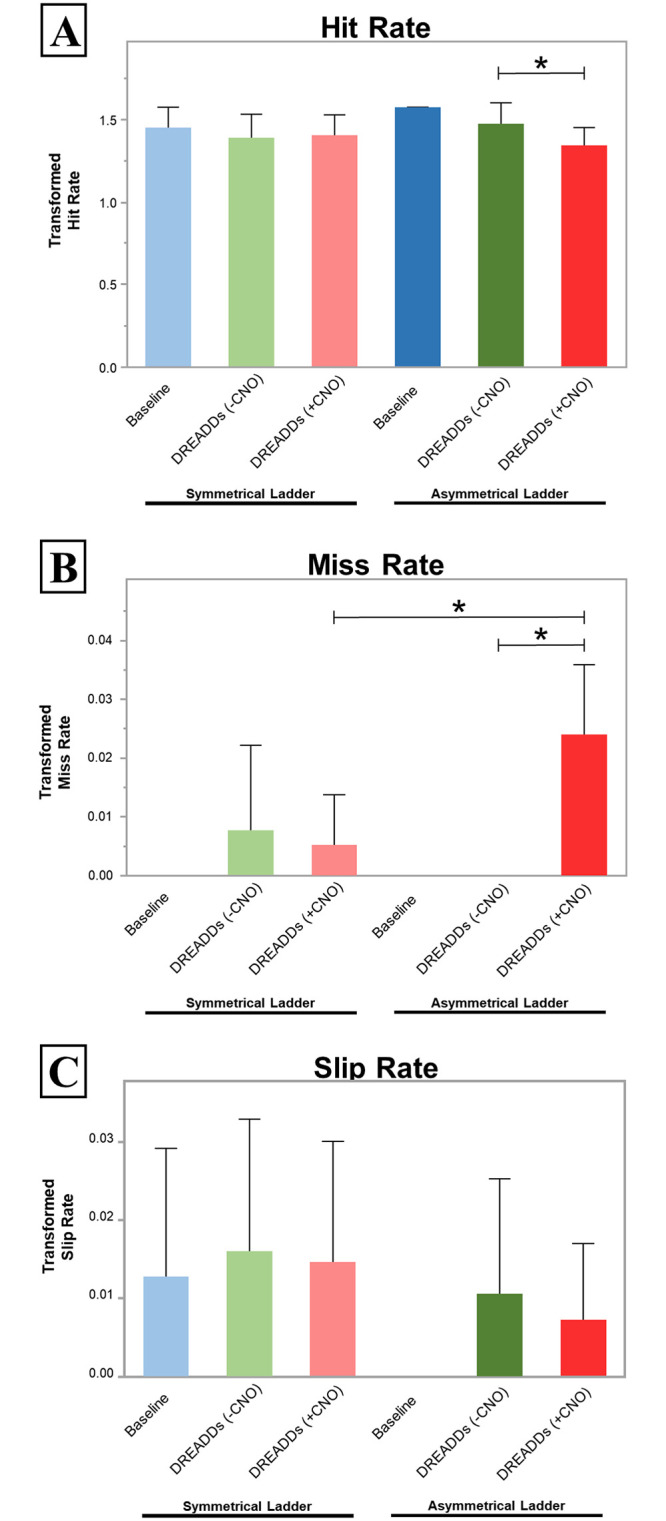
Locomotion data for each ladder type. (A) The arcsine transformed Miss rate of the right hindlimb symmetrical and asymmetrical ladder before DREADDs injections (Baseline), after DREADDs injection (–CNO), and after activation of DREADDs (+CNO). Significance was detected for the intra-ladder comparison between -CNO and +CNO conditions for the asymmetrical ladder only (p = 0.049). (B) The arcsine transformed Hit rate under the same conditions. Significance was detected in two conditions; the intra-ladder comparison between -CNO and +CNO conditions for the asymmetrical ladder (p = 0.000011), and the inter-ladder comparison for the +CNO conditions of the symmetrical and asymmetrical ladder (p = 0.0022). (C) The arcsine transformed Slip rate under the same conditions. We found no significant differences in percent Slip between any two conditions in either the symmetrical or asymmetrical rung arrangements. We observed no significant increases in percent Slip with DREADDs expression in any comparison. Asterisks denote significance at the p < 0.05 level. When not indicated, no significance was detected.

To evaluate the performance of our asymmetrical ladder relative to existing symmetrical systems, we performed ANOVA analysis comparing the same treatment conditions on the two ladder types. Henceforth, we refer to this analysis as “inter-ladder” comparison. Significance was not detected in hit, miss, or slip between any of inter-ladder “baseline” or inter-ladder “-CNO” conditions. However, a significant difference was observed comparing the “+CNO” condition between the symmetrical and asymmetrical ladders for misses (symmetrical (+CNO) miss rate = 0.0051 ± 0.00872, asymmetrical (+CNO) hit rate = 0.0239 ± 0.0118; *F* = 9.261; *df* = 5; p = 0.0022) (S4 Table). The complete description of all ANOVA results and comparisons considered significant below a p-value of 0.05 are show in S3 and S4 Tables for intra and inter-ladder comparisons respectively. Fig 4 shows the comprehensive results of arcsine transformed proportions for hits, misses, and slips on both ladder types for all three treatment conditions and indicates significance of comparisons for p < 0.05.

## Discussion

Sensitive probing of sensorimotor changes after disease or injury is crucial for basic neuroscience and evaluating potential therapies. Several instruments have been developed to quantify locomotor changes in rodents [2–4, 7–13]. One of these methods, the horizontal ladder walking task, is advantageous as it yields unambiguous and inexpensive assessment of locomotor recovery [2]. However, this task is limited to evaluating apparent differences in locomotion and may be unable to detect small changes [2–4]. This may be a result of cross limb transfer, a neural compensatory mechanism for estimation of an injured or perturbed limb’s position via information transferred from its contralateral pair [14]. The robust locomotion involved in cross limb transfer may conceal small sensorimotor changes that can be evoked by clinically relevant therapies (such as genetic neural manipulation), in the early onset of disease, or between higher levels of recovery. Unfortunately, few methodologies can currently provide sufficient sensitivity to identify small locomotor shifts, and ones that do are often prohibitively expensive (e.g. commercial motion capture systems). Here, we propose a novel ladder system—with angled rungs—as an additional tool to uncover subtle changes that may be overlooked by the standard ladder task. To evaluate our design, we examined ladder walking task performance of intact animals with hM3Dq DREADDs expressed in large diameter peripheral afferents. DREADD technology was chosen to validate our design as its subtle effects in the intact animal may be concealed by cross limb transfer [25].

Although existing ladder systems have shown adjustable positions, conventional horizontal ladders with equally or unequally spaced parallel rungs provide the same locomotion path for contralateral limb pairs. Similarly, other locomotor assays, such as beam walking tests and grid walk, do not force animals to adapt to different locomotion paths for contralateral limb pairs [11, 12]. The introduction of angled rungs in our asymmetrical ladder design uniquely decouples cross limb transfer by mimicking random perturbations that may occur in the native environment. Our results suggest that angled rungs are able to detect subtle locomotor changes by increasing the neural burden required of foothold selection, balance, and interlimb coordination.

Our asymmetrical design was developed to increase the sensitivity of the assay without dramatically decreasing its selectivity to false positives. The detection of significant inter-ladder changes and intra-ladder changes after DREADDs activation supports our hypothesis that a more complex design would more sensitively probe locomotor changes. Additionally, significant changes were not detected in baseline-baseline or (-CNO)-(-CNO) inter-ladder comparisons or in the baseline-(-CNO) intra-ladder comparisons. While we do not mean to imply the absence of the significance supports our hypothesis, we do claim that, in our hands, there is insufficient evidence to indicate that the asymmetrical design changes animal gait behavior unless intervention or injury is introduced. We believe this justifies further investigation into the use of complex horizontal ladders and other locomotor assays, such as grid walk, to elucidate minor or subtle changes in animal locomotion that may go undetected with existing designs.

While this study demonstrated that DREADDs expressed in the right lumbar dorsal root ganglia (DRG) influences locomotion in the intact animal, it does so subtly. We found significant differences in percent Hit and Miss when animals crossed asymmetrical rung arrangements during DREADDs activation. As lumbar large diameter afferents innervate muscles primarily about the hip and stimulation of afferents has been shown to extend joints [28], our data suggest enhanced neuronal firing by hM3Dq DREADDs may drive increased error rates by extending hindlimb joints. Further, we report an indetectable change in percent Slip. Our findings suggest that, of the targeted large diameter afferents, proprioceptive afferents may have been preferentially transduced. Since proprioceptive afferents are responsible for reporting information about limb position and limb dynamics while in motion to the central nervous system, it is reasonable that targeting of these afferents resulted in significantly increased hindlimb misplacement. Furthermore, if cutaneous afferents were not preferentially transduced, it is likely that an animal would be able to grasp appropriately as long as their hindpaw came in contact with a rung, which is consistent with our not detecting changes in hindlimb slippage.

Although significant, our findings illustrate the robustness of intact animal locomotion. This robustness may conceal locomotor shifts caused by increased neural excitation by hM3Dq DREADDs expressed in peripheral afferents. As such, standard rung arrangements may require additional subjects to elaborate DREADDs influence. Here, we show that angled rungs offer high sensitivity of detecting changes with a modest number of replicates, despite locomotor robustness, which, if broadly adopted, may aid other researchers in accomplishing the NIH guideline of reducing the number of subjects in animal experimentation [46].

Interestingly, in Baseline trials, animals crossed the asymmetrical ladder with complete accuracy, whereas they exhibited a small error rate on the symmetrical ladder. As with other irregularly spaced ladders [2], we changed rung arrangements between trial types (e.g. Baseline, DREADDs without CNO, and DREADDs activation) to prevent learning of rung patterns during repetitive trials. During testing, animals crossed the asymmetrical ladder at grossly observable lower velocities than the symmetrical ladder, regardless of CNO administration. This observation may be due to the higher neural burden of foothold selection of the asymmetrical design. As such, slower speeds may contribute to increased accuracy of footfalls during the task. While we noted that animals appeared to have slowed down, or were perhaps more conscientious, during asymmetrical ladder crossings, the quantitative evaluation of this occurrence is out of the scope of this study. Since animals accelerated, decelerated, and paused whilst locomoting across the horizontal ladder, time to completion does not provide indicators of the median speed. To rigorously examine speed differences between ladder types, high-speed kinematics would be required to quantify speed between footfalls on rungs.

One potential limitation of this study is the possibility that observed effects in locomotion were due to off-target effects of CNO inducing muscle dysfunction independent of our DREADDs transfection [47, 48]. While we believe this is unlikely to be the primary factor due to the positive visualization of our fluorescent markers (Fig 2D and 2E), doses below the known 5 mg/kg threshold, and the absence of expected observable behavioral changes in our observations, it is still possible that off-target CNO effects may have been a contributing factor in our results [47, 49]. However, if compounding off-target effects are present, the exclusive detection of significance in our asymmetrical ladder as compared to the symmetrical ladder should further bolster our claim to the sensitivity of our new assay.

Angled rungs are not only an attractive tool for investigation of locomotor changes after genetic manipulation, we speculate that they may also be able to detect deficits in the early onset of disease or to tease apart locomotor shifts during later stages of recovery. While genetic manipulation is of keen interest for therapeutic interventions after disease or injury, it is important to note that the biochemical mechanisms evaluated in this study are not likely observed in nature. As such, additional studies in a well-established preclinical model of neurological disorders wherein modest locomotor deficits are expressed will identify whether angled rungs are capable of distinguishing subtle locomotor differences over a longitudinal study. These data can be applicable in the administration of time-sensitive interventions.

The present study demonstrates the ability of angled rungs to elaborate error rates that may be overlooked by standard ladders by introducing different locomotion paths to contralateral limb pairs. Our asymmetrical ladder also has a high degree of flexibility, modularity, and reusability and can be applied to upgrade standard ladder locomotor analyses, including those for other animal models [50]. Our design enables for easy visualization of limb stepping and, as such, future additions to our design may also include adding multi-camera, high-speed motion capture kinematics. This could provide keen insight into differences in step kinematics during locomotion across standard and asymmetrical ladders that which are not grossly observable in standard ladder evaluation methods.

## Conclusion

We present an angled rung modification to the standard horizontal ladder walking task to increase the neural burden of foothold selection during the task. Angled rungs are able to achieve high sensitivity to detect subtle changes in locomotion by decoupling cross limb transfer. As such, our proposed design is a novel means to dissect changes in locomotion that may have been overlooked using standard horizontal ladders. Importantly, we believe our design can progress the current understanding of locomotor changes in the early onset of disease, between higher levels of recovery, or that occur by external manipulation.

## Supporting information

S1 Table Gap distances and angles by ladder type. (DOCX)

S2 Table Hit, miss, and slip values (group calculations). (DOCX)

S3 Table P-values of for intra-ladder ANOVA comparisons. (DOCX)

S4 Table P-values of for inter-ladder ANOVA comparisons. (DOCX)

S1 Appendix 2D CAD images used for laser cutting of the rung adjustment mechanism. (DWG)

S2 Appendix 3D rendering of the asymmetrical ladder. (ZIP)

S3 Appendix 3D rendering of the asymmetrical ladder. (ZIP)

S4 Appendix Symmetrical and asymmetrical ladder data. File contains raw data that was obtained from trained scorers.
(CSV)

S5 Appendix Hit, miss, and slip values for individual animals. Calculated totals, proportions (in percent), and arcsine transformed values for hits, misses, and slips per animal.
(CSV)
