# Correction: Addition of angled rungs to the horizontal ladder walking task for more sensitive probing of sensorimotor changes

**DOI:** 10.1371/journal.pone.0268603

**Published:** 2022-05-11

**Authors:** Jaclyn T. Eisdorfer, Michael A. Phelan, Kathleen M. Keefe, Morgan M. Rollins, Thomas J. Campion, Kaitlyn M. Rauscher, Hannah Sobotka-Briner, Mollie Senior, Gabrielle Gordon, George M. Smith, Andrew J. Spence

The following information is missing from the Funding statement: This work was supported by NIH grant 1R01NS114007-01A1.
