# Supplementary material for: Addition of angled rungs to the horizontal ladder walking task for more sensitive probing of sensorimotor changes
Source: PLoS One. 2021 Feb 5;16(2):e0246298. doi: 10.1371/journal.pone.0246298 (PMC7864417; doi:10.1371/journal.pone.0246298)
Supplement: S1 Table — (DOCX) [file pone.0246298.s001.docx]

**S1 Table. Gap distance and angle by ladder.**

| **Ladder Type** | **Average Gap** | **Minimum Gap** | **Maximum Gap** | **Average Angle** | **Minimum Angle** | **Maximum Angle** | **Total Rungs** |
| --- | --- | --- | --- | --- | --- | --- | --- |
| Symmetrical | 5.08 cm | NA | NA | 90° | NA | NA | 36 |
| Asymmetrical | 3.579 cm | 1.758 cm | 5.594 cm | 90.583° | 81.266° | 98.248° | 46 |

The average, minimum, and maximum values for the distance and angles between rungs on the symmetrical and asymmetrical ladder types and the total number of rungs used for each ladder configuration. All angles are measured perpendicular to the length of the ladder.
