# Supplementary material for: Addition of angled rungs to the horizontal ladder walking task for more sensitive probing of sensorimotor changes
Source: PLoS One. 2021 Feb 5;16(2):e0246298. doi: 10.1371/journal.pone.0246298 (PMC7864417; doi:10.1371/journal.pone.0246298)
Supplement: S2 Table — (DOCX) [file pone.0246298.s002.docx]

**S2 Table. Hit, miss, and slip values (group calculations).**

| **Evaluation** | **Ladder Type** | **Condition** | **Proportion (in %)** | **Arcsine Transformed** | **SD of Transformed Data** |
| --- | --- | --- | --- | --- | --- |
| Hit | Symmetrical | Baseline | 98.603682 | 1.448363 | 0.123570 |
|  |  | (-CNO) | 97.467995 | 1.387173 | 0.142193 |
|  |  | (+CNO) | 97.890514 | 1.401030 | 0.126080 |
|  | Asymmetrical | Baseline | 100.000000 | 1.570796 | 0 |
|  |  | (-CNO) | 98.843200 | 1.472332 | 0.125757 |
|  |  | (+CNO) | 96.815629 | 1.339500 | 0.111335 |
| Miss | Symmetrical | Baseline | 0 | 0 | 0 |
|  |  | (-CNO) | 0.779727 | 0.007799 | 0.014459 |
|  |  | (+CNO) | 0.510367 | 0.005104 | 0.008719 |
|  | Asymmetrical | Baseline | 0 | 0 | 0 |
|  |  | (-CNO) | 0 | 0 | 0 |
|  |  | (+CNO) | 2.392732 | 0.023931 | 0.011822 |
| Slip | Symmetrical | Baseline | 1.396318 | 0.013966 | 0.017286 |
|  |  | (-CNO) | 1.752278 | 0.017526 | 0.017600 |
|  |  | (+CNO) | 1.599119 | 0.015994 | 0.016157 |
|  | Asymmetrical | Baseline | 0 | 0 | 0 |
|  |  | (-CNO) | 1.156800 | 0.011570 | 0.015557 |
|  |  | (+CNO) | 0.791640 | 0.007917 | 0.010310 |

The hit, miss, and slip values for DREADDs animals and control cohorts of all conditions tested represented as a proportion (in percent) and the arcsine transformation of the proportion. The standard deviation of the arcsine transformed data is also listed. The conditions are as follows: Baseline – Prior to the administration of DREADDs. (-CNO) – Post-DREADDs administration without the CNO activator. (+CNO) – Post-DREADDs and including the CNO activator.
