# Supplementary material for: Addition of angled rungs to the horizontal ladder walking task for more sensitive probing of sensorimotor changes
Source: PLoS One. 2021 Feb 5;16(2):e0246298. doi: 10.1371/journal.pone.0246298 (PMC7864417; doi:10.1371/journal.pone.0246298)
Supplement: S3 Table — (DOCX) [file pone.0246298.s003.docx]

**S3 Table. Statistical values for intra-ladder ANOVA comparisons.** The P-values, F-values, and dfs calculated via ANOVA comparing rat hit, miss, and slip outcomes between different treatment conditions on the same ladders. Significance for a P < 0.05 is also listed. The conditions are as follows: Baseline – Prior to the administration of DREADDs. (-CNO) – Post-DREADDs administration without the CNO activator. (+CNO) – Post-DREADDs and including the CNO activator.

| **Comparison Type** | **Evaluation** | **Ladder Type** | **Condition** | **P-value** | **F-value** | **df** | **Significant?** |
| --- | --- | --- | --- | --- | --- | --- | --- |
| **Intra-ladder Comparison** | Hit | Symmetrical | Baseline-(-CNO) | 0.662626 | 0.421 | 4 |  |
|  |  |  | (-CNO)-(+CNO) | 0.978625 | 0.421 | 4 |  |
|  |  | Asymmetrical | Baseline-(-CNO) | 0.167522 | 10.03 | 4 |  |
|  |  |  | (-CNO)-(+CNO) | 0.048966 | 10.03 | 4 | * |
|  | Miss | Symmetrical | Baseline-(-CNO) | 0.315883 | 1.156 | 4 |  |
|  |  |  | (-CNO)-(+CNO) | 0.864019 | 1.156 | 4 |  |
|  |  | Asymmetrical | Baseline-(-CNO) | NA | NA | NA | NA |
|  |  |  | (-CNO)-(+CNO) | 0.000011 | 28.68 | 4 | * |
|  | Slip | Symmetrical | Baseline-(-CNO) | 0.91949 | 0.077 | 4 |  |
|  |  |  | (-CNO)-(+CNO) | 0.984506 | 0.077 | 4 |  |
|  |  | Asymmetrical | Baseline-(-CNO) | 0.138751 | 2.109 | 4 |  |
|  |  |  | (-CNO)-(+CNO) | 0.803427 | 2.109 | 4 |  |
