# Supplementary material for: Addition of angled rungs to the horizontal ladder walking task for more sensitive probing of sensorimotor changes
Source: PLoS One. 2021 Feb 5;16(2):e0246298. doi: 10.1371/journal.pone.0246298 (PMC7864417; doi:10.1371/journal.pone.0246298)
Supplement: S4 Table — (DOCX) [file pone.0246298.s004.docx]

**S4 Table. Statistical values for inter-ladder ANOVA comparisons**.

| **Comparison Type** | **Evaluation** | **Condition** | **P-value** | **F-value** | **df** | **Significant?** |
| --- | --- | --- | --- | --- | --- | --- |
| **Inter-ladder Comparison** | Hit | Baseline-Baseline | 0.915079 | 2.495 | 5 |  |
|  |  | (-CNO)-(-CNO) | 0.736482 | 2.495 | 5 |  |
|  |  | (+CNO)-(+CNO) | 0.368379 | 2.495 | 5 |  |
|  | Miss | Baseline-Baseline | NA | NA | NA | NA |
|  |  | (-CNO)-(-CNO) | 0.519439 | 9.261 | 5 |  |
|  |  | (+CNO)-(+CNO) | 0.002243 | 9.261 | 5 | * |
|  | Slip | Baseline-Baseline | 0.457845 | 0.297 | 5 |  |
|  |  | (-CNO)-(-CNO) | 0.968827 | 0.297 | 5 |  |
|  |  | (+CNO)-(+CNO) | 0.893545 | 0.297 | 5 |  |

The P-values, F-values, and dfs calculated via ANOVA comparing rat hit, miss, and slip between the two ladder types for the same treatment conditions. Significance for a P < 0.05 is also listed. The conditions are as follows: Baseline – Prior to the administration of DREADDs. (-CNO) – Post-DREADDs administration without the CNO activator. (+CNO) – Post-DREADDs and including the CNO activator.
